# Persistent genital ulcers due to herpes simplex virus and cytomegalovirus coinfection in a kidney transplant recipient

**DOI:** 10.1016/j.jdcr.2025.11.037

**Published:** 2025-12-18

**Authors:** Teeraphat Muangkham, Kajohnsak Noppakun, Romanee Chaiwarith, Siri Chiewchanvit, Mati Chuamanochan

**Affiliations:** a Division of Dermatology, Department of Internal Medicine, Faculty of Medicine, Chiang Mai University, Chiang Mai, Thailand; b Division of Nephrology, Department of Internal Medicine, Faculty of Medicine, Chiang Mai University, Chiang Mai, Thailand; c Pharmacoepidemiology and Statistics Research Center, Faculty of Pharmacy, Chiang Mai University, Chiang Mai, Thailand; d Division of Infectious Diseases and Tropical Medicine, Department of Internal Medicine, Faculty of Medicine, Chiang Mai University, Chiang Mai, Thailand

**Keywords:** cytomegalovirus, genital ulcers, herpes simplex virus, immunosuppression, kidney transplant

## Introduction

Genital ulcer disease, particularly in immunocompromised individuals, is most commonly caused by genital herpes simplex virus (HSV). The severity, chronicity, and recurrence of lesions are closely correlated with the extent and duration of immunosuppression.^1^ Coinfection with other viral pathogens, including cytomegalovirus (CMV), has also rarely been documented in individuals with advanced HIV infection or in solid organ transplant recipients undergoing immunosuppressive therapy.^2^, ^3^, ^4^, ^5^, ^6^, ^7^ We describe an uncommon case of concomitant CMV and HSV infection in a kidney transplant recipient. The patient presented with genital ulcers that were clinically indistinguishable from typical HSV lesions but failed to respond to standard antiviral therapy directed against HSV.

## Case presentation

A 61-year-old man with end-stage kidney disease who had undergone kidney transplantation 2 months previously was admitted with a 2-week history of multiple painful genital ulcers, intermittent fever, and chronic nonbloody diarrhea. His medical history included type 2 diabetes mellitus, hypertension, and dyslipidemia. His CMV serostatus was donor-positive/recipient-positive, and he received basiliximab as an induction therapy. His maintenance immunosuppressive regimen comprised tacrolimus (4 mg/d), mycophenolate mofetil (1000 mg/d), and prednisolone (5 mg/d). He had impaired allograft function with serum creatinine of 5.77 mg/dL. He was receiving prophylactic trimethoprim-sulfamethoxazole (400/80 mg, 4 tablets weekly) as prophylactic therapy. Acyclovir prophylaxis, initiated post-transplantation, was subsequently withheld due to leukopenia, which was suspected to be multifactorial, with acyclovir considered a lower-priority agent within the regimen.

**Fig 1:**
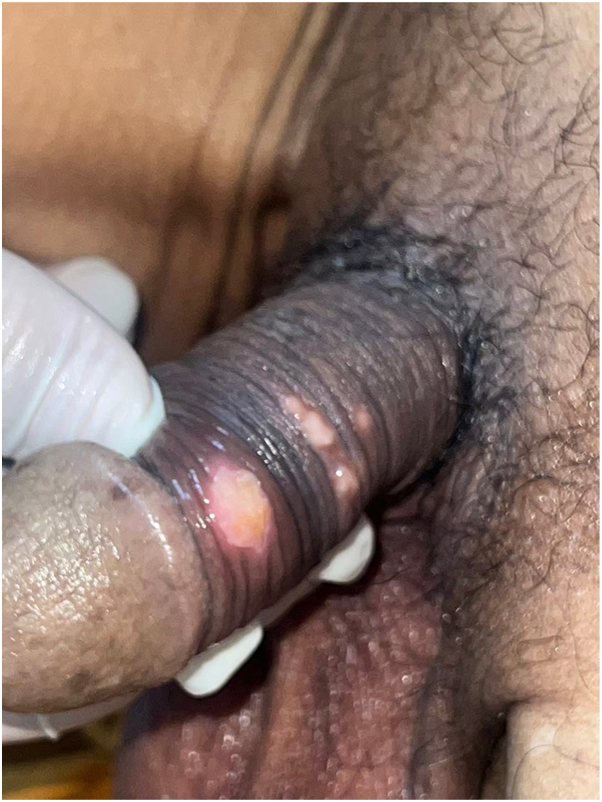
Multiple well-demarcated, clean-based ulcers on the penile shaft.

**Fig 2:**
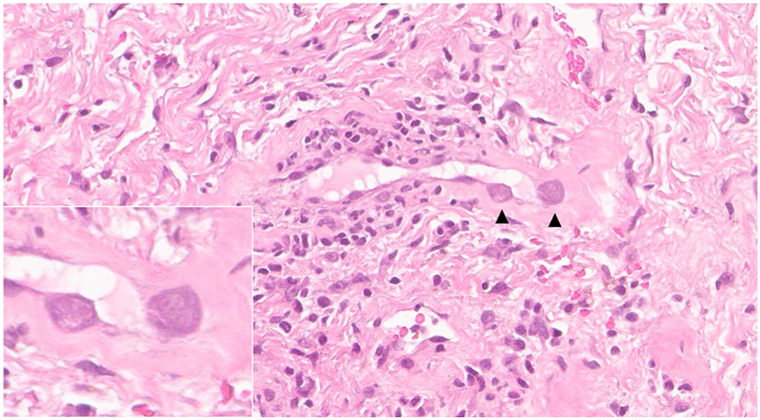
Histopathologic examination of a skin biopsy demonstrates markedly enlarged endothelial cells containing *large*, basophilic intranuclear inclusions with a surrounding clear halo and smaller cytoplasmic inclusions (*black arrow head*) (hematoxylin-eosin stain, ×400).

**Fig 3:**
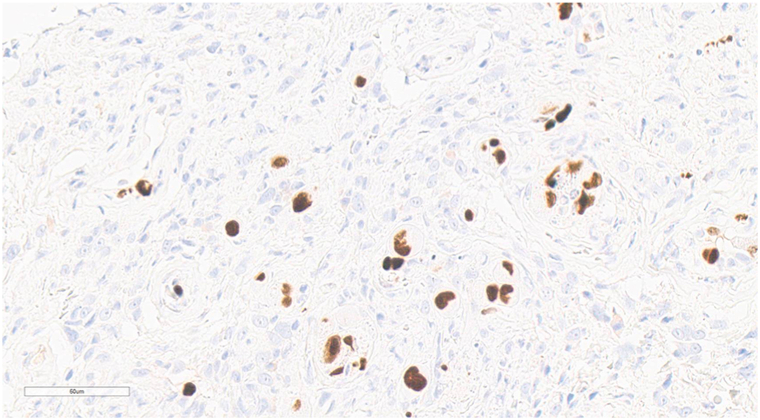
Immunohistochemical staining for monoclonal antibody directed against CMV highlights multiple positively stained cells (*brown chromogen*), confirming CMV involvement (IHC, ×400). *CMV*, Cytomegalovirus; *IHC*, immunohistochemistry.

Physical examination revealed multiple well-demarcated, clean ulcers on the penile shaft (Fig 1). No oral lesions, ocular symptoms, or cutaneous eruptions were observed. The patient was febrile but hemodynamically stable. Acute kidney injury from hypovolemia improved with hydration, and the white blood cell count was normal. Dermatologic consultation identified multinucleated giant cells on Tzanck smear obtained from the ulcer base. Oral acyclovir (200 mg twice daily) was initiated for presumed HSV infection, appropriately adjusted for renal function. After 7 days, the ulcers persisted, and the white blood cell count remained stable. Ulcer swab polymerase chain reaction was positive for HSV-2 with a high viral load (6.45 log IU/mL); HSV-1 was not detected. A skin biopsy was performed due to inadequate clinical response, which revealed mixed inflammatory infiltrates accompanied by markedly enlarged endothelial cells exhibiting prominent basophilic intranuclear inclusions with perinuclear clearing, along with smaller associated cytoplasmic inclusions (Fig 2). Immunohistochemical staining of the skin biopsy specimens confirmed the presence of CMV infection (Fig 3).

During hospitalization, the patient developed exertional dyspnea. CMV pneumonia was diagnosed based on positive polymerase chain reaction of bronchoalveolar lavage fluid, with a serum CMV load of 339,989 IU/mL (5.53 log IU/mL). Intravenous ganciclovir was initiated, leading to clinical improvement and resolution of ulcers within 2 weeks. CMV colitis was suspected as the cause of diarrhea, but colonoscopy was deferred after spontaneous resolution. Mycophenolate mofetil was discontinued in the setting of active systemic infection and subsequently replaced with everolimus at a dosage of 1 mg/day to reduce the overall burden of immunosuppression while maintaining adequate graft protection.

Following a full 2-month course of ganciclovir therapy, the serum CMV viral load decreased to below the threshold of quantification, measured at <31 IU/mL (<1.49 log IU/mL), and ganciclovir treatment was discontinued. The patient was discharged in stable clinical condition with close outpatient follow-up arranged for continued monitoring.

## Discussion

CMV typically affects visceral organs, including the gastrointestinal tract, lungs, and retina. However, cutaneous involvement, while relatively uncommon, may manifest in immunocompromised patients, particularly in individuals with severely diminished cell-mediated immunity, such as those with CD4+ T-lymphocyte counts <50/μL or recipients of intensive immunosuppressive regimens.^1^ These lesions most frequently present as painful, well-demarcated anogenital ulcers, often with associated necrosis or ulcerative erosions, and may coexist with concurrent HSV-related disease.^1^^,^^5^ In solid organ transplant recipients, CMV reactivation represents a well-recognized complication, with peak incidence typically occurring within 4 to 12 months following transplantation.^4^^,^^6^^,^^8^ Although cutaneous manifestations of CMV infection are uncommon relative to visceral involvement, their presence may serve as a clinically relevant early indicator of systemic viral dissemination, particularly in high-risk populations.^1^^,^^4^^,^^7^ Coinfection with CMV and HSV in cutaneous tissues has been well documented in immunocompromised hosts, including transplant recipients and individuals with advanced HIV infection, wherein CMV may potentiate the severity of HSV-induced lesions, contribute to ulcer persistence, and delay mucocutaneous healing.^1^^,^^5^

A definitive diagnosis of CMV-associated genital ulcerations requires histopathological confirmation. Typical findings include enlarged endothelial or stromal cells with prominent intranuclear inclusions surrounded by a clear halo—the classic “owl’s eye” morphology. These inclusions are pathognomonic of active viral replication. To enhance diagnostic accuracy, immunohistochemical staining for CMV antigens is considered the gold standard, particularly in equivocal cases or when hematoxylin-eosin staining is inconclusive.^4^^,^^5^^,^^8^

The management of CMV-related cutaneous and mucocutaneous lesions depends on disease severity and the degree of immunosuppression. Therapy should be individualized, considering the patient’s immune status, transplant history, and potential antiviral resistance. First-line treatment is systemic ganciclovir or its oral prodrug valganciclovir, both inhibiting viral DNA polymerase. In cases of resistance, adverse effects, or intolerance, alternatives such as foscarnet or maribavir serve as second-line options. Valganciclovir and letermovir are more frequently used in the context of prophylaxis, particularly among solid organ or hematopoietic stem cell transplant recipients, to mitigate the risk of CMV reactivation in high-risk individuals.^9^^,^^10^ Letermovir acts uniquely by inhibiting the CMV DNA terminase complex.^10^ For patients with suspected HSV coinfection, ganciclovir usually suffices as monotherapy due to its dual coverage against CMV and HSV.

This case highlights the importance of considering viral coinfection in refractory genital ulcers, particularly in immunocompromised patients. When ulcers persist despite adequate HSV-directed therapy, CMV should be suspected, especially in advanced HIV infection or post-transplant settings.^6^, ^7^, ^8^ In these contexts, impaired immune surveillance increases the risk of systemic reactivation, and mucocutaneous lesions may serve as early indicators of disseminated disease. Recognition of such cutaneous signs can prompt timely diagnosis and treatment, potentially improving outcomes in high-risk populations.

### Declaration of generative AI and AI-assisted technologies in the writing process

During the preparation of this work, the authors used ChatGPT (GPT-5 Mini, OpenAI) in order to enhance language clarity and improve academic style. After using this tool, the authors reviewed and edited the content as needed and take full responsibility for the content of the publication.

## Conflicts of interest

None disclosed.
